# Stability of the timing of food intake at daily and monthly timescales in young adults

**DOI:** 10.1038/s41598-020-77851-z

**Published:** 2020-11-30

**Authors:** Andrew W. McHill, Cassie J. Hilditch, Dorothee Fischer, Charles A. Czeisler, Marta Garaulet, Frank A. J. L. Scheer, Elizabeth B. Klerman

**Affiliations:** 1 Division of Sleep and Circadian Disorders, Departments of Medicine and Neurology, Brigham and Women’s Hospital, 221 Longwood Ave, Boston, MA 02115 USA; 2 Division of Sleep Medicine, Department of Medicine, Harvard Medical School, 221 Longwood Ave, Boston, MA 02115 USA; 3 Department of Sleep and Human Factors Research, Institute for Aerospace Medicine, German Aerospace Center, Cologne, Germany; 4 Department of Physiology, University of Murcia, 30100 Murcia, Spain; 5 Present Address: Oregon Institute of Occupational Health Sciences, Oregon Health and Science University, 3181 SW Sam Jackson Park Road, Portland, OR 97239 USA; 6 Present Address: Fatigue Countermeasures Laboratory, Department of Psychology, San José State University, San José, CA 95192 USA; 7 Present Address: Department of Neurology, Massachusetts General Hospital, 100 Cambridge St, 20th Floor, Boston, MA 02114 USA

**Keywords:** Risk factors, Nutrition, Circadian rhythms and sleep

## Abstract

Cross-sectional observations have shown that the timing of eating may be important for health-related outcomes. Here we examined the stability of eating timing, using both clock hour and relative circadian time, across one semester (n = 14) at daily and monthly time-scales. At three time points ~ 1 month apart, circadian phase was determined during an overnight in-laboratory visit and eating was photographically recorded for one week to assess timing and composition. Day-to-day stability was measured using the Composite Phase Deviation (deviation from a perfectly regular pattern) and intraclass correlation coefficients (ICC) were used to determine individual stability across months (weekly average compared across months). Day-to-day clock timing of caloric events had poor stability within individuals (~ 3-h variation; ICC = 0.12–0.34). The timing of eating was stable across months (~ 1-h variation, ICCs ranging from 0.54–0.63), but less stable across months when measured relative to circadian timing (ICC = 0.33–0.41). Our findings suggest that though day-to-day variability in the timing of eating has poor stability, the timing of eating measured for a week is stable across months within individuals. This indicates two relevant timescales: a monthly timescale with more stability in eating timing than a daily timescale. Thus, a single day’s food documentation may not represent habitual (longer timescale) patterns.

## Introduction

Weight gain and obesity rates have continued to rise globally^1,2^, despite the efforts of public health initiatives designed to combat this epidemic^3^. College-aged individuals have the highest rates of increased weight gain compared to the general population or any other age group^4–6^. Weight gain during this time may have major public health implications as college is a critical developmental time for establishing long-term health behaviors^7,8^. As overweight or obese adolescents have double the risk for high weight status in adulthood^9^, identification of mechanisms and behaviors by which college students are vulnerable to weight gain, and thus could be targeted by countermeasures, is imperative to combat this global obesity epidemic.

Mounting evidence suggests that the timing of eating plays an important role in cardiometabolic health^10–15^. We have shown that, relative to lean individuals, non-lean individuals consume the majority of their daily calories later, specifically closer to their dim-light melatonin onset (DLMO), a circadian phase marker denoting the start of a person’s biological night^16,17^. Other reports have also found relationships between later eating and weight gain^18^ and higher body mass index^19^. However, these observations of the timing of eating and poor health have come from short, cross-sectional protocols using clock time (which is not a physiological variable) as the reference point. The reliability of cross-sectional studies of meal timing, and more specifically the circadian timing (i.e., a physiological variable) of caloric intake, to accurately reflect the timing of eating has been largely unexplored.

The stability of meal intake (in addition to its timing) has also been hypothesized to play a role in cardiometabolic health^20^. Often, meal stability is discussed as the regular consumption (or skipping) and size of specified meals (breakfast, lunch, dinner, and snacks) or the number of meals, across multiple days. In these cases, less stable patterns of eating timing have been shown to be associated with poorer cardiometabolic health^20–22^. These reports^20–22^ on meal stability are also limited in that stability is typically examined only across several days within a week, and not multiple time-points further apart. By measuring regularity as either consuming or not consuming a meal, rather than examining day-to-day stability in the timing of caloric events (defined as any time calories are consumed > 15 min apart^16,23,24^), as we have previously done using day-to-day stability of sleep metrics^25^, important information regarding stability may be overlooked.

We examined the stability of the timing of food consumption, referenced to both clock time and relative to circadian time, at three time points across a semester in college undergraduates both for all individuals and for lean vs non-lean group. Specifically, we tested the hypotheses that (i) the timing of food consumption would be stable across multiple months, and (ii) the physiologically-based circadian timing of food consumption would be more stable than clock timing of food consumption. Additionally, we tested the exploratory hypotheses that (iii) individuals with a lean body composition would have more stable timing of food consumption and number of daily calories per month compared to non-lean individuals. It is critical to understand these relationships in order to determine whether results from cross-sectional studies can be meaningfully extrapolated into potential predictors for disease or used in countermeasures to combat poor health and disease, and encourage healthy eating behaviors.

## Materials and methods

### Participants

Participants (n = 15) were studied during one spring semester at one US university. One lean participant (male) did not complete any meal diaries and was excluded from the analyses; thus 14 individuals (5 female), aged 19.1 years (± standard error of mean (SEM), range; 0.3, 18–21) with an average body mass index of 22.9 kg/m^2^ (0.8, 18.3–28.5), were studied. To be eligible, participants needed to: have a device capable of downloading the food tracking application; not be currently employed in night shift work; and not have traveled more than one time zone in the 3 months prior to and during the protocol. Study procedures were approved at the Brigham and Women’s Hospital by the Partner’s Healthcare Institutional Review Board (#2012P001631) and performed in accordance to the Declaration of Helsinki. All participants provided written informed consent. This trial was registered at clinicaltrials.gov as NCT02846077. Note, only a subset of participants from the overall trial partook in this semester-long study, and no participants or their data from previous publications^16,17,25^ were used in the current study.

### Field study procedures

To track meals throughout the three separate occasions across the protocol, participants used the photographic mobile phone application MealLogger (Wellness Foundry, New York, NY) for 7-consecutive days (including both school/work and free days). Each food monitoring week occurred near the time of each in-laboratory visit; none occurred during the in-laboratory visit and it was random as to whether it occurred before or after the visit and day of the week that it started. All food monitoring weeks occurred when participants were at school; none was a vacation week. The food monitoring app allowed participants to take a time-stamped photograph of all food and beverages consumed, label what meal was being consumed (breakfast, lunch, dinner, snack, or beverage) and write a detailed description of the meal content (i.e., any ingredients or foodstuffs unable to be identified from the photo)^16,17^. Participants were asked to include an object of known size within the picture to help calculate portion size and to take a second photo if the meal was not fully consumed to estimate total caloric intake. After participants had uploaded the picture and description of their meals, the photos were available to study nutritionists and study staff for scoring of timing of meal, caloric content, and macronutrient composition. Participants were also able to take a picture for later upload if out of internet range or cell-service at the time of the meal. If any aspect of the photo was unclear, or if the participant documented ≤ 2 meals within a waking day, staff followed up with participants via email or through the mobile app within 24 h after upload.

### In-laboratory procedures

Participants were admitted to the Brigham and Women’s Hospital Center for Clinical Investigation and Intensive Physiologic Monitoring Unit at the beginning, middle, and end of semester (i.e., three separate occasions) for an approximate 16-h overnight stay to assess DLMO timing as a marker of circadian timing and to determine body composition. Beginning at ~ 15:30 h, ambient lighting was lowered to dim settings (~ 4 lx) and saliva samples were collected hourly beginning at ~ 16:00 h and ending at ~ 07:00 h (16 samples per participant) and later assayed for melatonin. Upon admission, participants were not allowed to use any personal light-emitting electronic devices, and were instructed to refrain from eating or drinking and to maintain a constant seated posture for 20 min immediately prior to each saliva sample collection, to minimize any potential exogenous influences on melatonin concentrations. At all other times during the protocol, participants were able to eat a provided snack, ambulate within the study room, remain seated, or sleep in a seated position. If participants slept, they were awakened by research staff immediately prior to saliva collection.

During each visit to the laboratory, participants also had their body composition measured via four-lead bioelectrical impedance (Quantum II BIA analyzer, RJL Systems, Clinton Township, MI); all metals and devices were removed and measurements were conducted on participants in the supine position. Impedance measurements were performed three times per visit and an average impedance score of the three measures per visit was used for analysis.

### Analysis

DLMO was determined as the linear interpolated point in time at which melatonin concentrations crossed and remained above a 5 pg/ml threshold^26^. All caloric events were assigned a circadian phase relative to the timing of each participant’s DLMO (which was assigned 0°).

The timing of each caloric event record was reviewed within 24 h of entry and double checked for accuracy by a second staff member. Caloric content and macronutrient compositions of each entry were independently scored by two research dieticians within the Brigham and Women’s Hospital Center for Clinical Investigation using the University of Minnesota Nutrition Data System for Research software^27,28^. Any caloric event that was recorded ≤ 15 min of a previous event and labeled as the same type of meal (e.g., lunch) was combined with the previous entry into one ‘caloric event’^16,23^. The caloric midpoint was calculated as the time at which 50% of each individuals’ daily calories were consumed^16^ and peak calories as the circadian phase each day in which participants consumed their highest caloric intake, both measured daily and averaged across each week.

To assess day-to-day stability of meal timing, the Composite Phase Deviation (CPD) metric was applied to the first, midpoint, and last daily caloric event. The CPD combines two components: (i) how different meal timing is compared to that on the previous day (regularity component) and (ii) how far away it is from the daily mean (alignment component)^29^. CPD scores reflect the average deviation in hours from a perfectly regular pattern of meal timing (i.e., same time every day).

$$\begin{gathered} \Delta {\text{Regularity}}_{{\text{i}}} { }(\Delta {\text{DD}}_{{\text{i}}} ) = {\text{Meal timing}}_{{{\text{i}} - 1}} - {\text{Meal timing}}_{{\text{i}}} \hfill \\ \Delta {\text{Alignment}}_{{\text{i}}} { }(\Delta {\text{AT}}_{{\text{i}}} ) = {\text{Average meal timing}} - {\text{ Meal timing}}_{{\text{i}}} \hfill \\ {\text{CPD}}_{{\text{i}}} = \sqrt {\Delta {\text{DD}}_{{\text{i}}}^{2} + \Delta {\text{AT}}_{{\text{i}}}^{2} } \hfill \\ {\text{CPD}} = { }\frac{1}{{\text{N}}}\mathop \sum \limits_{{{\text{i}} = 1}}^{{\text{N}}} {\text{CPD}}_{{\text{i}}} \hfill \\ \end{gathered}$$

where DD stands for “day-to-day”, AT for “average timing”, and i denotes a given day and N the total number of days. The metric was originally developed for sleep timing but has been previously applied to the daily timing of social events^25^. We also computed the standard deviation of first, midpoint, and last caloric event for each individual for each week of measurement.

In order to account for physiological sex differences in body fat compositions, sex-dependent criteria were used to separate participants into lean (n = 8, 1 female) or non-lean (n = 6, 4 female) body composition groups. Non-lean participants were defined as an average body fat percentage across the three in-lab visits ≥ 31% for females and ≥ 21% for males^30^; lean participants had an average body fat percentage below those criteria.

All variables were first analyzed using mixed-effects models (variance components) with month (each individuals’ 7-day monitoring average within month 1, 2, or 3) as a categorical fixed factor and participant as a random factor to determine group differences across months. If a main effect was present, planned comparison dependent t-tests were used to determine differences between months and Bonferroni corrections were applied (p < 0.017 needed to reach significance) to correct for multiple comparisons. To examine individual consistency of each variable within a participant across measurement months, intra-class correlation coefficients (ICC) were used to determine the stability of individual differences across months for a single score, two-way mixed-effects model^31^. The strength of ICC scores were defined using the following criteria: slight (0.00–0.20), fair (0.21–0.40), moderate (0.41–0.60), substantial (0.61–0.80), and almost perfect (0.81–1.00)^32^. Relationships between months were also analyzed using Pearson correlations after being measured for normality using skewness and kurtosis metrics. Statistical analyses were performed using SAS 9.4 (SAS Institute Inc., Cary, NC).

## Results

On average, participants were studied across 104 days (overall 3-month average ± SEM, range for 3 months; 2.4, 80–112 days) during the semester. Data collection (identified as Months 1–3) occurred at weeks 2–6 (month 1), 7–11 (month 2), and 12–16 after start of overall study (month 3). Across the semester, average overall DLMO timing was 23:26 (0:29, 20:06–03:32), which did not significantly differ between months (Table 1). To explore the relationship between the stability of metrics and body composition, we analyzed the associations between months in individuals separated into lean (n = 8) and non-lean (n = 6) categories based on sex-specific body fat percentage criteria^30^. To first understand whether differences in melatonin stability were driven by body composition, we tested the relationship between DLMO timing between months in each group. We found a significant positive association in DLMO timing in the non-lean group between month 1 and 2 (r = 0.94, p < 0.01) and in both lean (r = 0.9, p = 0.04) and non-lean (r = 0.93, p < 0.01) groups between months 1 and 3; there were no significant associations between month 1 and 2 in the lean group (r = 0.78, p = 0.07).

**Table 1:** Means (SEM) and mixed model main effects of month for eating metrics.

Metric	Month 1	Month 2	Month 3	F-value	p-value
**Circadian**					
DLMO (hh:min)	23:20 (00:25)	23:40 (00:33)	23:24 (00:44)	0.25	0.78
**Caloric timing**					
Breakfast (hh:min)	10:07 (00:18)	11:09 (00:30)	10:37 (00:33)	3.37	0.05
Lunch (hh:min)	14:27 (00:30)	14:34 (00:27)	14:01 (00:22)	0.55	0.58
Dinner (hh:min)	19:35 (00:20)	19:47 (00:27)	19:20 (00:17)	0.60	0.56
Snack (hh:min)	15:53 (00:56)	18:29 (00:48)	19:54 (01:16)	4.13	**0.04**
First caloric event (hh:min)	10:52 (00:35)	12:22 (00:35)	11:38 (00:41)	5.50	**0.01**
Caloric midpoint (hh:min)	15:17 (00:32)	16:24 (00:40)	16:07 (00:50)	1.98	0.16
Last caloric event (hh:min)	19:18 (00:32)	20:11 (00:48)	19:58 (00:36)	1.04	0.37
**Circadian caloric timing**					
Calories within 4 h of DLMO to sleep onset (%)	21.7 (4.3)	27.1 (4.6)	27.3 (5.2)	0.04	0.96
Phase of peak caloric timing (°)	229.9 (8.8)	238.6 (10.1)	242.6 (7.3)	1.09	0.35
Caloric midpoint to DLMO difference (h)	− 8.1 (0.6)	− 7.2 (0.6)	− 7.2 (0.5)	0.98	0.39
**Day-to-day variability**					
CPD first caloric event (h)	2.7 (0.3)	3.3 (0.5)	3.1 (0.6)	1.08	0.36
CPD caloric midpoint (h)	3.6 (0.4)	3.4 (0.5)	4.1 (0.5)	0.71	0.50
CPD last caloric event (h)	4.8 (0.4)	5.3 (0.5)	4.7 (0.5)	0.55	0.59
Std first caloric event (h)	2.0 (0.2)	2.4 (0.4)	2.1 (0.4)	0.73	0.49
Std caloric midpoint (h)	3.5 (0.3)	3.7 (0.3)	3.3 (0.3)	0.42	0.66
Std last caloric event (h)	2.7 (0.3)	2.4 (0.4)	2.7 (0.3)	0.41	0.67
**Caloric consumption**					
Daily average calories (kcal)	1664.8 (121.5)	1485.4 (131.5)	1827.8 (180.1)	3.78	**0.04**
Daily average meals (number)	2.9 (0.2)	2.8 (0.4)	3.2 (0.4)	0.99	0.37
Daily average fats (% total energy)	36.0 (1.6)	35.5 (2.1)	33.7 (2.4)	1.02	0.38
Daily average carbohydrates (% total energy)	46.8 (2.3)	47.1 (2.9)	49.1 (2.9)	1.13	0.34
Daily average proteins (% total energy)	17.1 (1.1)	17.3 (1.2)	17.2 (1.6)	0.05	0.95

Values are means (SEM). P-values represent mixed model comparisons across months. Bolded values represent significant (p < 0.05) differences. *DLMO* dim-light melatonin onset, *CPD* composite phase deviation, *std* standard deviation.

Note that average first caloric event may be later than breakfast due to how caloric events were labelled by the individual (e.g., if the individual “skipped” breakfast but still had a snack). Likewise, the last caloric event may be earlier than dinner if dinner was not consumed (e.g., snack eaten in the afternoon could be last caloric event).

### Day-to-day variability in timing of eating

To compute day-to-day variability in the timing of food consumption, we applied the CPD (i.e., average deviation in hours from a perfectly regular pattern of meal timing) and calculated standard deviation for the first, midpoint, and last daily caloric events for each participant every day within each week. In general, across the duration of study, the CPD for the first daily caloric event was 3.1 h (0.4, 0.8–5.3), midpoint was 3.7 h (0.4, 1.2–5.5), and last was 4.9 h (0.3, 3.0–6.7), whereas standard deviations were 2.2 h (0.2, 0.2–5.1), 3.5 h (0.2,1.1–6.0) and 2.6 h (0.2, 0.4–5.7), respectively; there were no significant main effects for month for any CPD or standard deviation metric (all p > 0.36, Table 1). CPD metrics were relatively inconsistent within participants across months, with ICC values ranging from slight (ICC = 0.12 and 0.13 for first daily caloric event and caloric midpoint, respectively) to fair (ICC = 0.34 for last daily caloric event) in strength. Standard deviations ranged from slight (ICC = 0.18 for caloric midpoint) to moderate (ICC = 0.43 for both first and last daily caloric events). There was a significant positive association in the CPD for first daily caloric event and standard deviation of caloric midpoint between months 1 and 3 in the lean group (r = 0.96, p < 0.01; r = 0.95, p < 0.05), but no significant relationships between months in any other CPD or standard deviation metric for either lean (all p > 0.12) or non-lean (all p > 0.11) groups.

### Stability of eating behaviors by clock timing

To determine the stability of eating timing across months, data were analyzed using mixed-effects models across months. At this population level, there were no significant effects of month across the study in weekly timing of participant-identified breakfast, lunch, or dinner; there was a significant main effect of month for snacks (Table 1). The variation across months was ~ 1 h (in comparison to the ~ 3 h day-to-day variation detailed above). Planned comparisons of snack timing did not reveal any significant differences between specific months (all p > 0.06). There was overlap in the timing of breakfast and lunch meals, and of snack meals and all other meals. We also calculated the weekly average group timing of the first daily caloric event, the time at which 50% of daily calories were consumed (i.e., caloric midpoint), and the last daily caloric event. There was a significant main effect of month for the timing of the first daily caloric event, but not the caloric midpoint or the last daily caloric event (Table 1), with the first caloric event significantly earlier during month 1 versus month 2 (p < 0.01).

**Figure 1:**
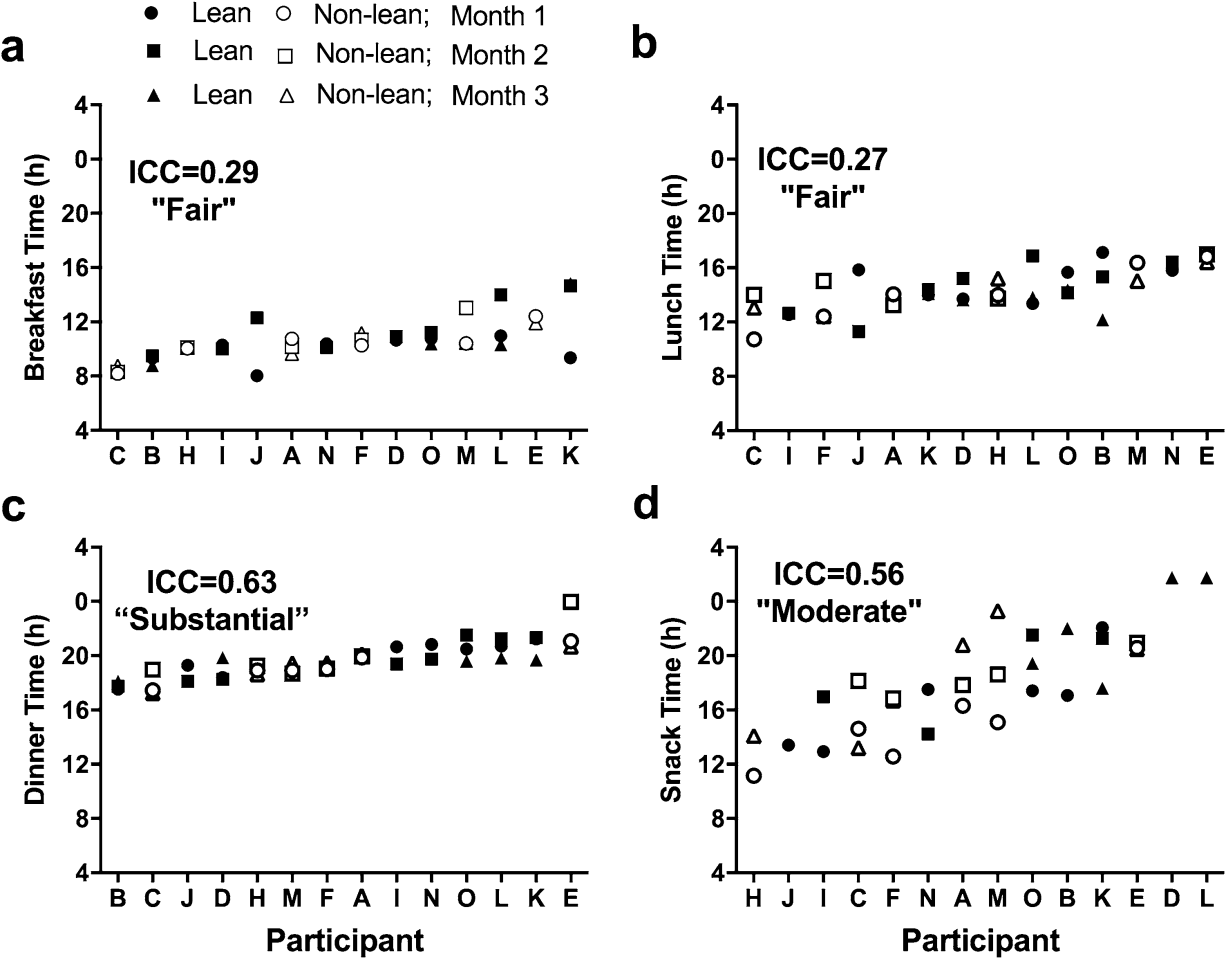
Individual differences in breakfast (**a**), lunch (**b**), dinner (**c**) and snacks (**d**) timing during three different months across the semester. Participants are ordered from earlier to later timing for each measure. Closed symbols denote lean participants and open denote non-lean participants; circles denote month 1, squares month 2, and triangles month 3. Intraclass correlation coefficients (ICC) with the strength of the scores are presented on each panel.

**Figure 2:**
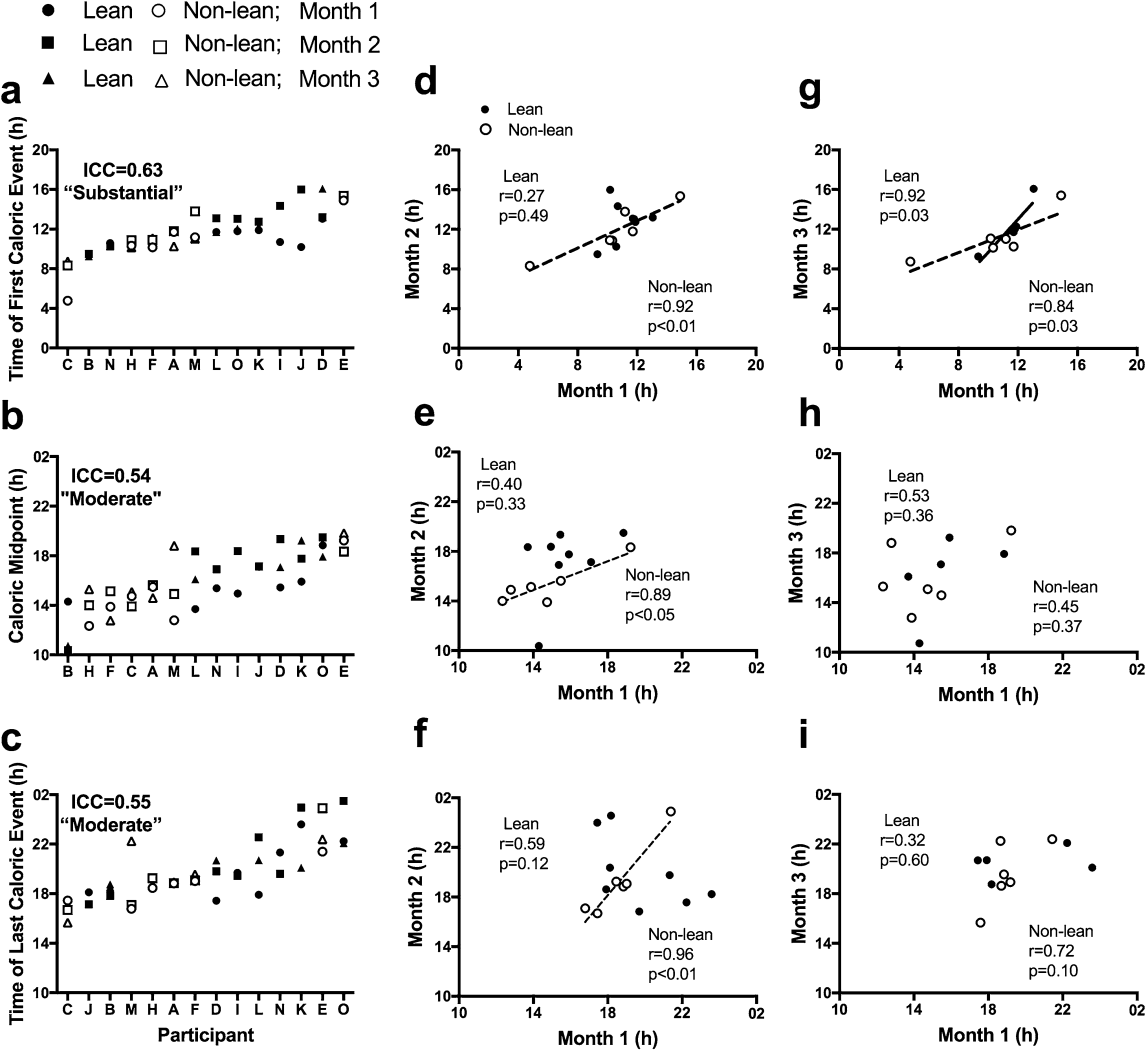
Individual differences in the clock time of the first, midpoint, and last caloric events during three different months (**a**–**c**) and relationship between timing of caloric events and months (**d**–**i**). Participants are ordered from earlier to later timing for each measure. Closed symbols denote lean participants and open denote non-lean participants; circles denote month 1, squares month 2, and triangles month 3. Intraclass correlation coefficients (ICC) with the strength of the scores are presented on each panel. Solid and dashed-lines represent significant correlations between months in lean and non-lean participants, respectively.

At the individual level across the three months, participants were least consistent in the timing of breakfast and lunch (ICC score of fair relationship strength), more consistent in snack timing (moderate relationship strength), and most consistent in dinner timing (substantial relationship strength) (Fig. 1). Participants tended to be more individually consistent in the timing of the first (substantial relationship strength), than in the midpoint and last caloric event (both moderate relationship strengths) (Fig. 2a–c).

### Influence of body composition on stability of eating behaviors relative to clock timing

When comparing the lean and non-lean participants’ meal timing between months, there was a significant positive association for dinner timing in both lean (r = 0.84, p = 0.01) and non-lean (r = 0.83, p = 0.04) groups and for snack timing in the non-lean group (r = 0.94, p = 0.02) between months 1 and 2. There were also significant positive associations when comparing months 1 and 3 for breakfast (r = 0.85, p = 0.03), lunch (r = 0.86, p = 0.03), and dinner (r = 0.94, p < 0.01) in the non-lean group; there were no significant associations when comparing month 1 and 2. When examining consistency between months in the timing of first, midpoint, and last daily caloric events, we found significant positive associations for the timing of first, midpoint, and last daily caloric events in the non-lean group between months 1 and 2 and a significant positive association in the timing of first daily caloric event between months 1 and 3 for both groups (all p ≤ 0.05, Fig. 2d–i).

### Stability of eating behaviors by circadian timing

**Figure 3:**
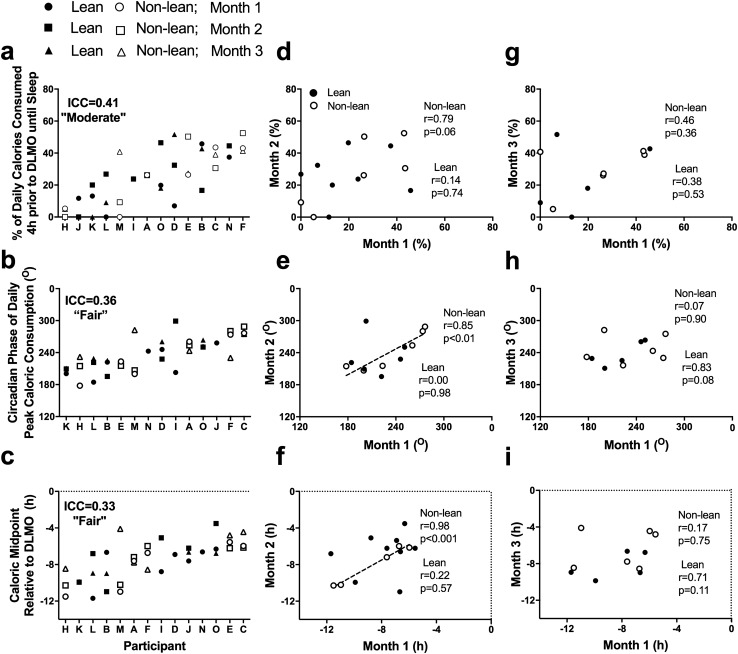
Individual differences in the circadian timing of caloric events during three different months (**a**–**c**) and relationship between circadian timing of caloric events and months (**d**–**i**). Participants are ordered from earlier to later timing for each measure. Closed symbols denote lean participants and open denote non-lean participants; circles denote month 1, squares month 2, and triangles month 3. Intraclass correlation coefficients (ICC) with the strength of the scores are presented on each panel. Solid and dashed-lines represent significant correlations between months in lean and non-lean participants, respectively. DLMO, dim-light melatonin onset. A circadian phase of 0° denotes timing of DLMO.

To examine meal timing in relation to a physiological marker, the timing of caloric events was analyzed relative to DLMO. We first analyzed meals within 4 h of DLMO until sleep onset as any caloric events eaten during that time would likely result in a postprandial energetic response that would continue after DLMO^33^ and to be consistent with our prior work^16^. At the group level, there were no significant effects of month across the study for: the percentage of calories consumed within 4 h of DLMO until sleep onset; the circadian phase of peak caloric timing; or the timing of caloric midpoint relative to DLMO (Table 1). Nonetheless, when examining the relationship on an individual level, the ICC score for percentage of calories consumed within 4 h of DLMO until sleep onset had a moderate relationship strength, whereas the ICC scores for circadian phase of peak caloric timing and the timing of caloric midpoint relative to DLMO both had a fair relationship strength (Fig. 3a–c).

### Influence of body composition on stability of eating behaviors relative to circadian timing

There were no significant associations in lean and non-lean participants when examining consistency between months for the percentage of calories consumed within 4 h of DLMO until sleep onset (both p > 0.06). For the circadian phase of peak caloric timing and the timing of caloric midpoint relative to DLMO, however, we observed significant positive associations between months 1 and 2 in the non-lean group, but not the lean group (Fig. 3d–i).

### Stability of caloric and macronutrient intake

On average, our study participants consumed 1627 kcal (127, 840–2346) daily across the three months during the study, with a significant effect of month (Table 1). However, there were no significant differences in daily calories consumed between particular months by planned comparisons (all Bonferroni-corrected p > 0.03). These calories were consumed in an average of 2.9 meals (0.3, 1.7–6.4) per day, with no significant month effect for meal number. In regards to macronutrients consumed, there were no significant main effects for percentage of daily calories from fat, carbohydrates, or protein consumed at the group level across months (Table 1).

**Figure 4:**
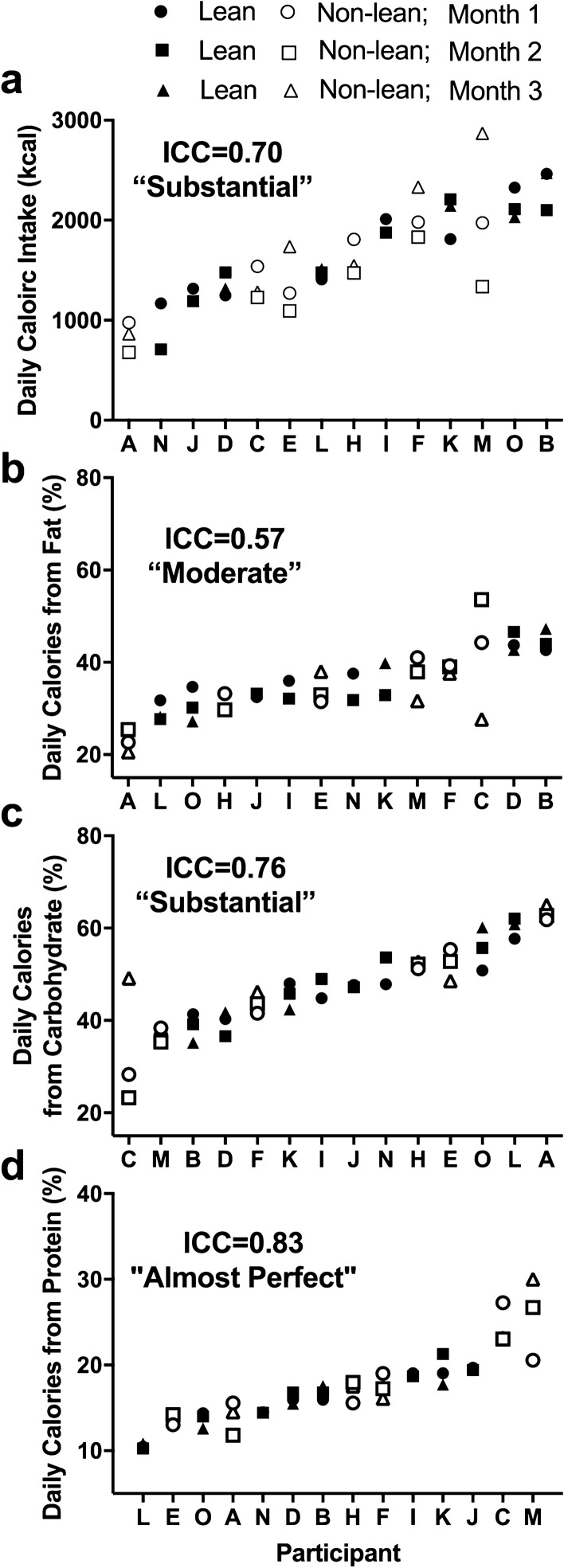
Individual differences in daily caloric intake (**a**), percent of daily calories from fat (**b**), carbohydrates (**c**), and proteins (**d**) during three different months. Participants are ordered from lower to higher amounts for each measure. Closed symbols denote lean participants and open denote non-lean participants; circles denote month 1, squares month 2, and triangles month 3. Intraclass correlation coefficients (ICC) with the strength of the scores are presented on each panel.

Individuals were relatively consistent across months for caloric intake (Fig. 4). In particular, we found that the strength of the ICC relationships ranged from moderate (percent of daily calories from fat) to substantial (daily calories and percent of daily calories from carbohydrates) to almost perfect (percent of daily calories from protein) (Fig. 4). There was no intra-individual consistency between macronutrients (e.g., consistency in percent of fat intake was not always associated with consistency of carbohydrate intake). In terms of meal number, we found that individuals were moderately consistent across months, with a significant positive association between number of meals in months 1 and 2 (r = 0.90, p = 0.002) and months 1 and 3 (r = 0.89, p = 0.04) for lean participants, but not for non-lean participants (r = 0.75, p = 0.09 and r = 0.28, p = 0.59, respectively).

## Discussion

Determining the reproducibility, or stability, of behaviors significantly associated with optimal health from cross-sectional findings is fundamental to the scientific process. The current study reveals that although there were significant differences in several of our variables of interest at the month level, the individual stability of timing of eating in these participants living in real-world settings was fair-to-almost perfectly stable when measured across a week and compared across 3-months of observation relative to clock time. Moreover, day-to-day timing of eating was only slight-to-moderately consistent at the individual level, which may suggest two relevant time scales: a monthly timescale with more stability in eating timing than a daily timescale. Taken together, these data suggest that week-long cross-sectional studies regarding eating behaviors are likely reflecting habitual behaviors at the individual level, and that a single day’s food documentation may not represent habitual (i.e., longer time-scale) behaviors.

Unique to this study, we analyzed the consistency of meal timing across multiple months of observation, both as clock time and in relation to circadian time. Interestingly, the strength of individual stability tended to be strongest when clock timing of caloric events (i.e., first, midpoint, and last caloric event) were analyzed rather than participant identified meals. It was expected that the timing of caloric events would fare better than self-identified meals, as caloric events are unaffected by a skipped or misidentified meals. This point is highlighted by the fact that the participants’ average first caloric event tended to be later than a meal identified as breakfast, potentially signifying a skipping of breakfast. We suggest that future studies using timing of any caloric intake rather than timing of a meal as the appropriate metric.

Interestingly, clock timing of caloric events was more stable than caloric events in relation to circadian timing. One explanation for these findings could be external constraints (i.e., a social component, short amount of time, other obligations) of when a participant may choose to eat their primary meals (i.e., breakfast, lunch, and dinner)^34^. For example, because snacking is often done alone and meals are more commonly consumed with others^35^, the timing of when a social network is available to eat together on a daily basis could drive meal timing and decrease stability in eating relative to circadian time. We have recently shown that students attending the same college can have wide ranges of DLMO timing (> 10 h)^17^, and thus if meal timing is partially influenced by social constructs, two participants with large differences in DLMO choosing to eat together would decrease stability relative to circadian time but not clock time. The fact that the timing of dinner and the last daily caloric events were similar may support this point, as individuals may choose to eat with others as opposed to eating alone later at night. Despite this explanation, and other exogenous causes of variability, it is important to note that the stability of meal timing was always at least fair in strength. Future work is needed in identifying the consistency of meal timing relative to circadian timing outside of the college setting and also how social constructs may influence the timing of eating.

In addition to examining the stability of meal timing at an individual level, we also performed an exploratory analysis of the stability of meal timing when participants were separated into lean and non-lean body composition groups. Counter to our initial hypothesis, we found that non-lean individuals tended to have higher stability in timing of meals and caloric events between months (significant correlations in 11 out of 30 of our timing metrics) as compared to the lean group (4 out of 30 timing metrics). Because we found that our non-lean group had more stable DLMO timing across months, this could help account for the more regular timing of calories relative to circadian timing. This was unexpected as previous accounts have found less stable eating patterns associated with higher body fat compositions and poorer health^20–22^. One potential explanation for our differences in findings may be how stable meal intake is defined. Often, stability is described as the number of meals consumed within a day; this was not how we defined it here. A more variable number of meals consumed day-to-day, or frequency of meals, tends to be associated with poorer health^21,36,37^.

Our finding that day-to-day stability across months within an individual was somewhat poor, as compared to the average timing of caloric events, might reflect a general reliability issue for metrics of stability^38^. Mean values may have good reliability with few measurements, whereas variability measures (e.g., standard deviation) often need a high number of repeated observations to achieve reasonable reliability (i.e., as many as 50^39^). Thus, an increased observation sample may help to improve day-to-day stability metrics. Another potential problem with single day assessments of dietary behaviors may be altered behaviors associated with the recording of those behaviors; this change in behaviors is less likely with longer recording durations.

Although our protocol spanned over an entire semester, with three separate highly controlled inpatient overnight-visits, this study is not without limitations. First, our low sample size limited our ability to draw conclusions for some lean vs. non-lean metrics, for non-college or other college populations, and about the timing of meals and cardiometabolic health. Previously, we have shown that the circadian timing of caloric intake is associated with higher body fat composition^16^. In the current study, we were under-powered to perform any regression analysis in regard to meal timing and body composition metrics, and thus could not test to replicate our previous findings. Second, although we intentionally studied participants across the duration of a semester, inherent changes associated with a progressing semester (e.g., exams, seasonal changes, etc.) may have led to exogenous influences impacting our outcome measures. Moreover, by recording data across 7-consecutive days during each measurement interval, we may have increased day-to-day variability due to caloric events potentially being later on free-days as compared to school/work days. However, we would argue that these exogenous influences due to real-life demands actually strengthen our findings as they demonstrate how robust the stability of these behaviors are despite outside influence and allow for real-world generalizability of our findings. Future work randomizing or starting cohorts at serial times and including populations that are not college students are still needed to fully elucidate the repeatability of our metrics.

In summary, our findings document the stability of the timing of caloric consumption in relation to both clock hour and the physiological circadian timing. Our findings suggest that food intake timing of eating is fairly stable across multiple months within individuals, even though day-to-day variability of the timing of eating within a week in individuals has poor stability, suggesting that a single day’s food documentation may not represent habitual behaviors. Lastly, our surprising findings that non-lean individuals tended to have a higher stability of meal timing, and the relation between stability, body composition, and cardiometabolic health needs further examination.

## Data Availability

Data are archived on Brigham and Women’s Hospital servers and available upon request.
